# Supplementary material for: The interactome of intact mitochondria by cross-linking mass spectrometry provides evidence for coexisting respiratory supercomplexes
Source: Mol Cell Proteomics. 2017 Dec 8;17(2):216–32. doi: 10.1074/mcp.RA117.000470 (PMC5795388; doi:10.1074/mcp.RA117.000470)
Supplement: Supplemental Data [file supp_RA117.000470_133922_0_supp_23978_fzffwf.zip › spectra_annotation/mito_DR_spectra_annotation/412-1-4-1-5-1.pdf]

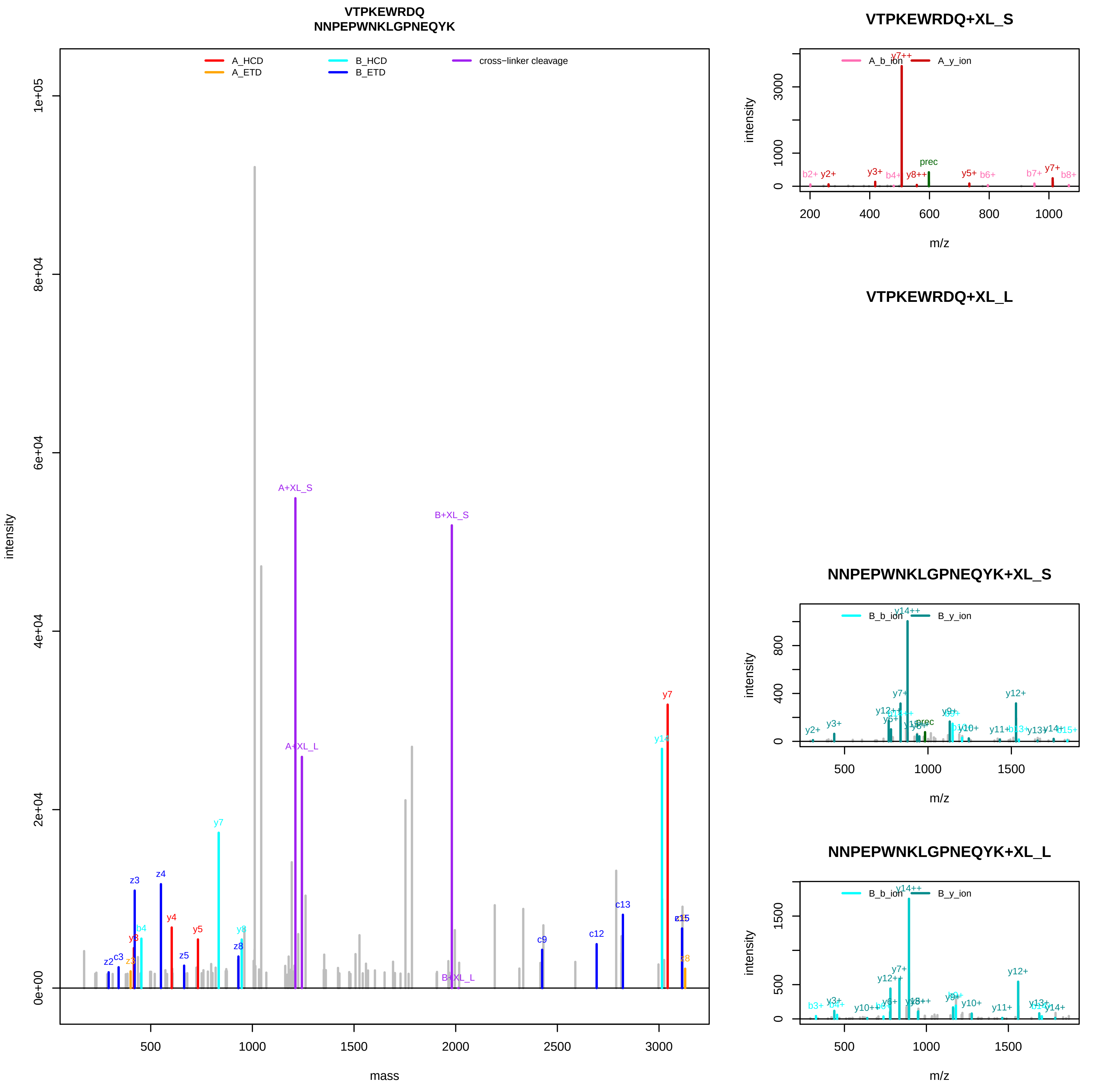
VTPKEWRDQ
NNPEPWNKLGPNEQYK
A_HCD
A_ETD
B_HCD
B_ETD
cross−linker cleavage
A+XL_S
B+XL_S
A+XL_L
B+XL_L
y7
y14
z15
z8
c13
c12
c9
z2
c3
z3
y3
b4
z4
y4
y5
z5
z8
y8
intensity
mass
0e+00
2e+04
4e+04
6e+04
8e+04
1e+05
500
1000
1500
2000
2500
3000
VTPKEWRDQ+XL_S
A_b_ion
A_y_ion
y7++
prec
b2+
y2+
y3+
b4+
y8++
y5+
b6+
b7+
y7+
b8+
0
1000
3000
200
400
600
800
1000
m/z
VTPKEWRDQ+XL_L
NNPEPWNKLGPNEQYK+XL_S
B_b_ion
B_y_ion
y14++
y7+
y12+
y12++
y9+
y2+
y3+
y10+
y11+
y13+
y14+
b15+
0
400
800
500
1000
1500
m/z
NNPEPWNKLGPNEQYK+XL_L
B_b_ion
B_y_ion
y14++
y7+
y12++
y12+
b3+
y3+
b4+
y10++
y6+
y9+
y10+
y11+
y13+
y14+
0
500
1500
500
1000
1500
m/z
